# Supplementary material for: In vivo Serial Passaging of Human–Simian Immunodeficiency Virus Clones Identifies Characteristics for Persistent Viral Replication
Source: Front Microbiol. 2021 Nov 18;12:779460. doi: 10.3389/fmicb.2021.779460 (PMC8636705; doi:10.3389/fmicb.2021.779460)
Supplement: Supplementary file 1 [file Data_Sheet_1.PDF]

Supplementary Table 1. Synonymous mutations observed in HSIV-P3 IMCs

Gene	HSIV-P3-114	HSIV-P3-161	HSIV-P3-284
GAG	S9S, R58R, E208E, S234S		S9S,
POL	E184E, K508K, R999A	A78A, E184E, K508K, T627T	E184E, E379E, K508K, C780C
VIF	V10V, L109L, Q130Q	V10V, L109L, Q130Q	V10V, L109L, Q130Q
VPU	R36R, P75P		
ENV	V85V, 178K, T230T, G522G, L690L, V810V	V85V, 178K, T448T, A556A, L690L, V810V	V85V, 178K, L690L, V810V
NEF		G96G	

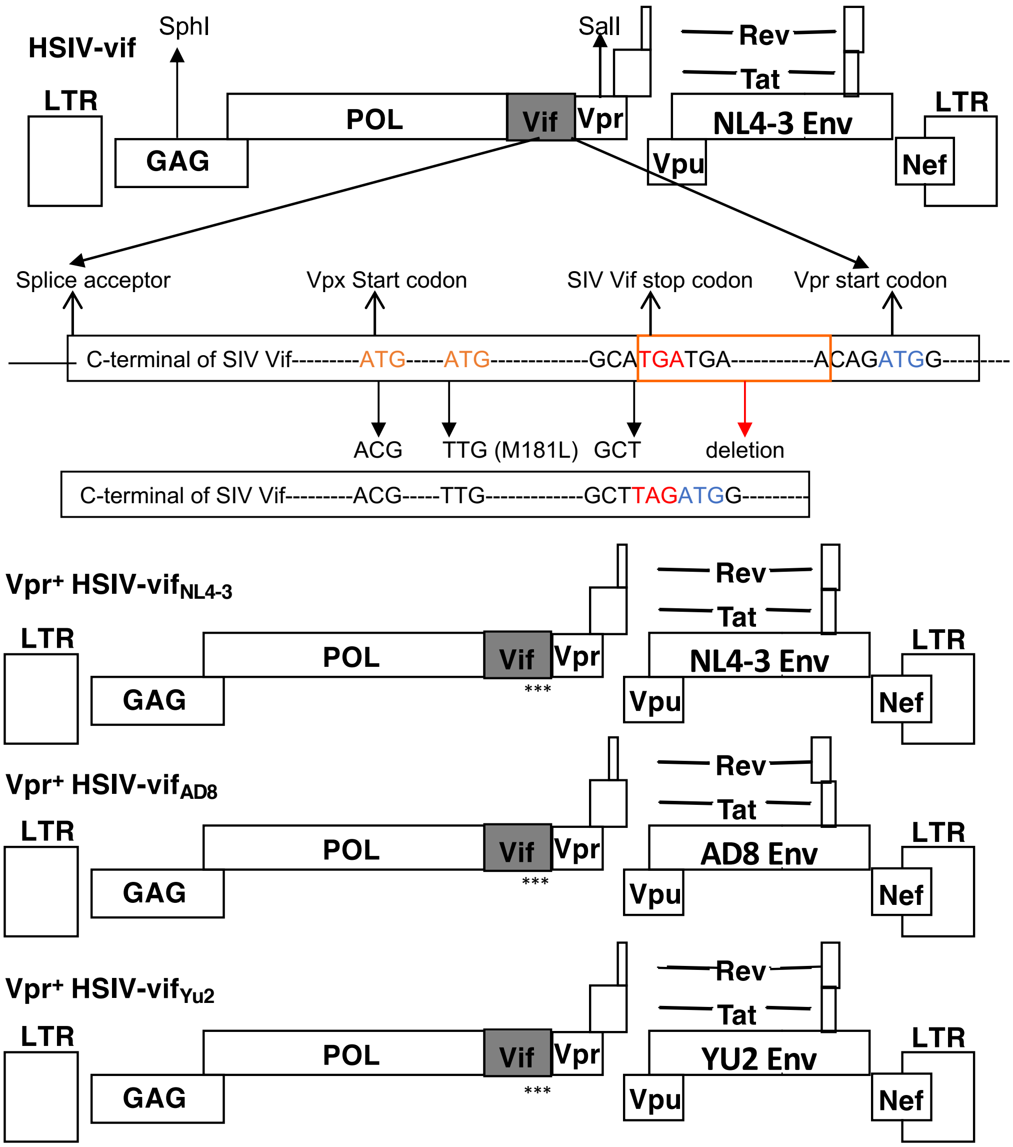

Supplementary Fig. 1: Construction of Vpr expressing HSIV-vif clones. SphI to SalI fragment of HSIV-vif$_{NL4-3}$ encompassing HIV *gag, pol,* SIV *vif* and HIV-1 vpr genes was cloned into pCR2.1 TOPO vector. SIV *vpx* start codon and two additional ATG codons upstream of the HIV-1 *vpr* start codon were mutated by Quickchange mutagenesis and the sequence between the stop codon of *vif* and start codon of *vpr* were deleted. After mutagenesis, SphI and SalI fragment was cloned back into HSIV-vif$_{NL4-3}$ and HSIV-vif$_{AD8}$. Similarly, SphI to SalI fragment of HSIV-vif$_{Yu2}$ was cloned into pCR2.1 TOPO vector, ATG codons upstream of *vpr* were mutated, and cloned back into HSIV-vif$_{Yu2}$. *Approximate location of mutations introduced in the *vif* gene.

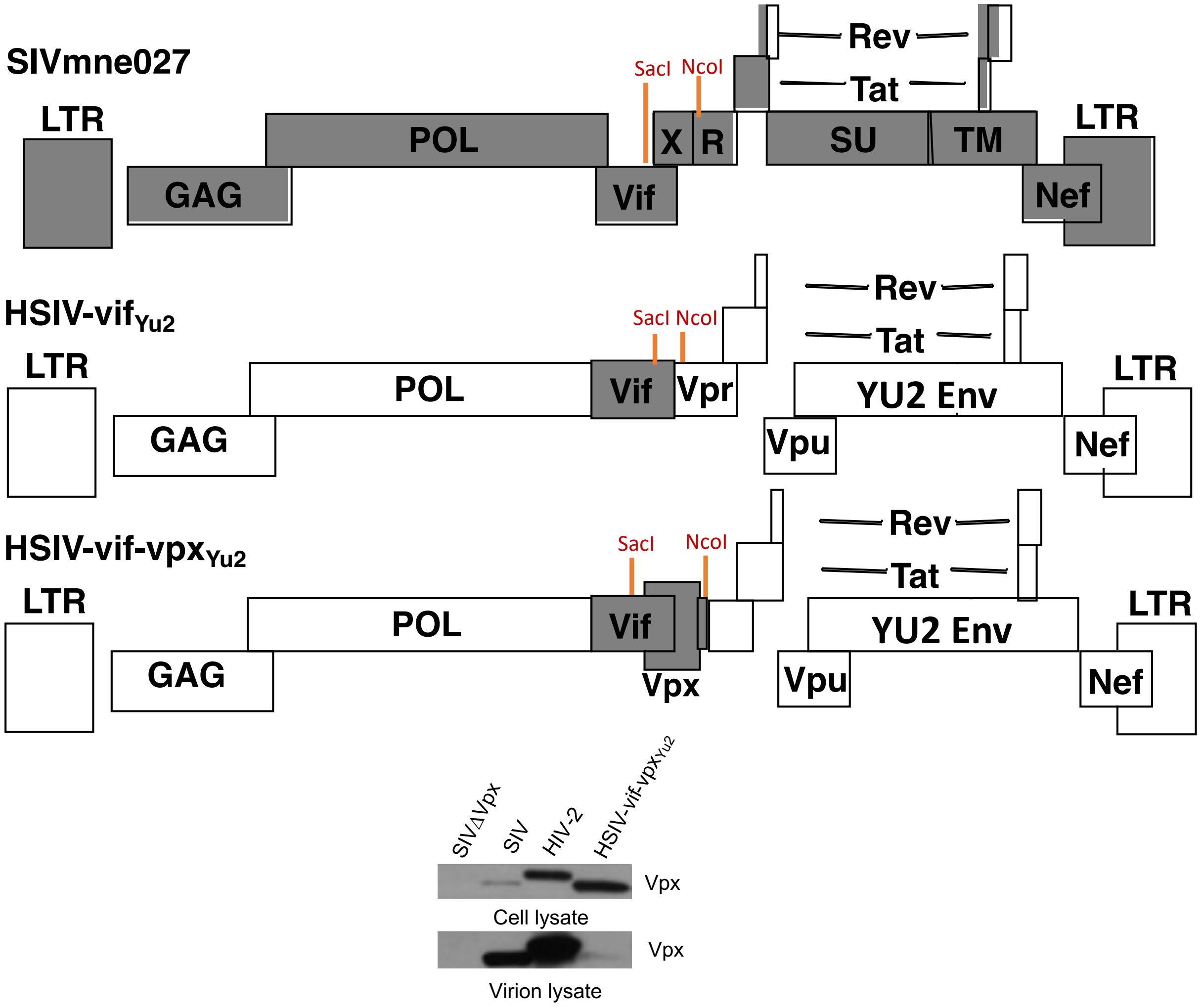

Supplementary Fig. 2: Construction of Vpx expressing HSIV-vif $_{YU2}$. SacI to NcoI region of HSIV-vif$_{Yu2}$ was replaced with SacI to NcoI region of SIVmne027 to generate HSIV-vif-vpx$_{Yu2}$. 293T cells were transfected with different lentiviral plasmids. At 48 hours post-transfection, virus supernatants were collected and concentrated by centrifugation. Cell and virion lysates were analyzed by western blot using antibody to HIV-2 Vpx protein.

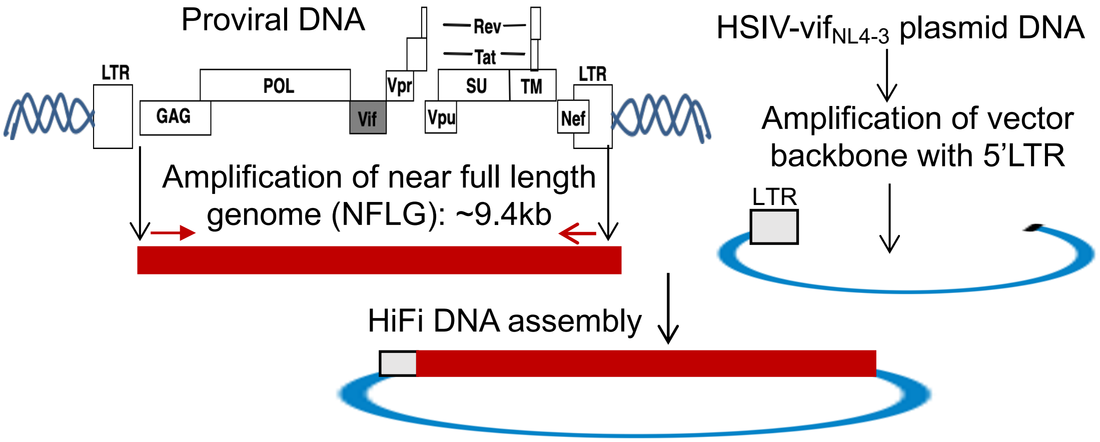

Supplementary Fig. 3: Schematic of HiFi DNA assembly approach to generate full length clones. Near full length genomes (NFLG) were amplified using nested PCR and cloned into a vector PCR product containing 5' LTR sequences (amplified from HSIV-vif$_{NL4-3}$ plasmid) using NEBuilder HiFi assembly mix.

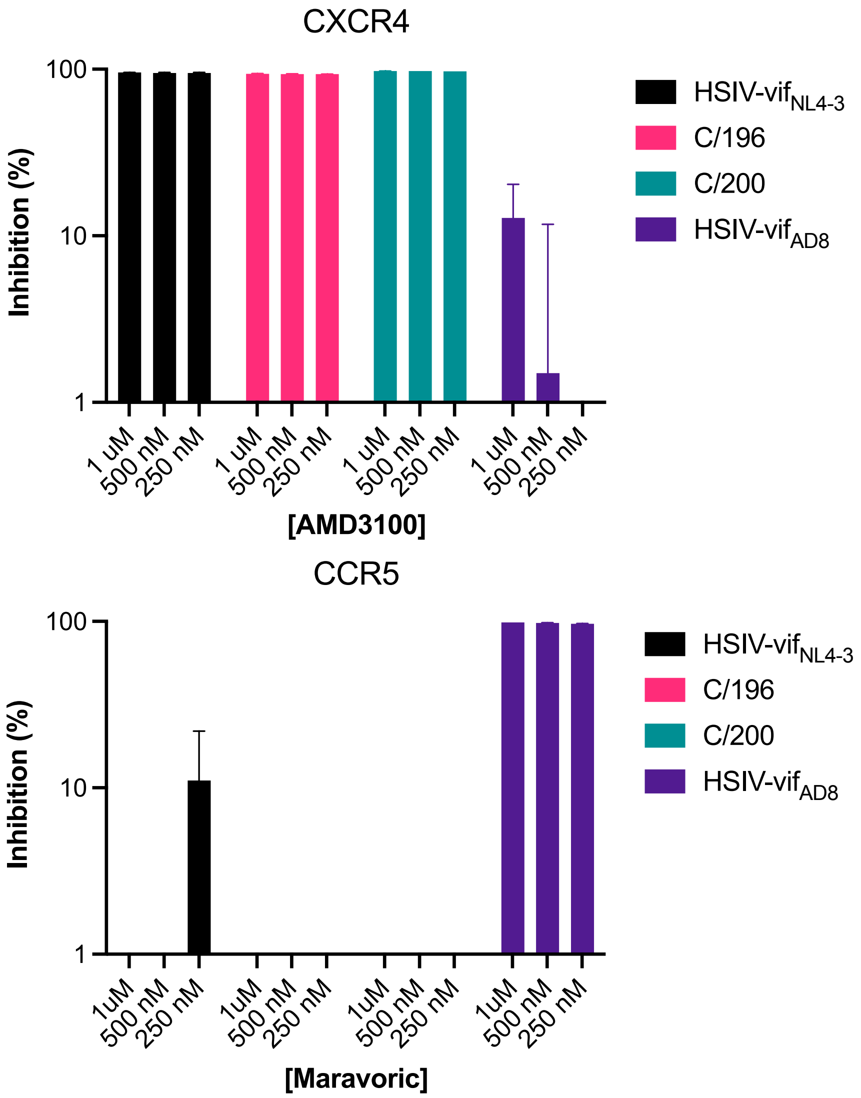

Supplementary Fig. 4: Coreceptor usage of biological clones C/196 and 200-2. TZM-bl cells were infected with different viruses in the presence of increasing concentrations of CXCR4 and CCR5 inhibitors. 48 hours later cell lysates were assayed for luciferase activity using plate based luminometer.

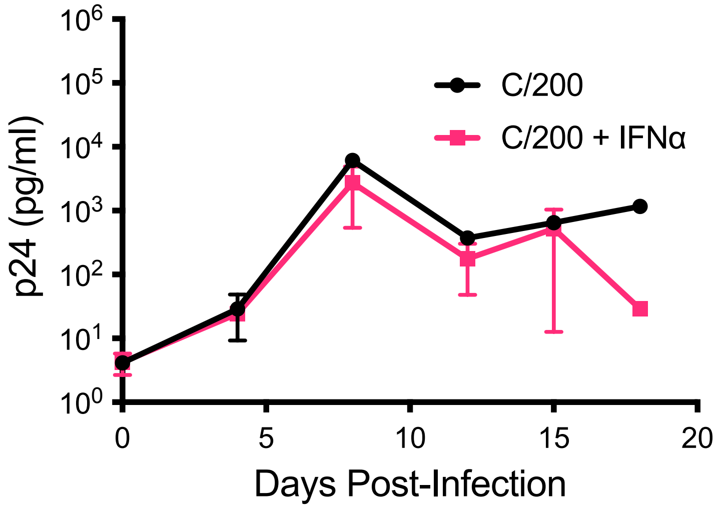

Supplementary Fig. 5: Replication kinetics of biological isolate of HSIV-vif$_{NL4-3}$ (C/200). PTM CD4+ T cells were infected in duplicate at a MOI of 0.01 with C/200 in the presence or absence of IFNα (200 U/ml) in the culture media. Virus supernatants were collected every 3 to 4 dpi and p24 was quantified by ELISA.

Nef sequences:

```
HSIV-vif NL4-3     1 MGGKWSKSSVIGWPAVRERMR-RAEPAADGVGAVSRDLEK 39
C/196              1 ....................RA................. 40
C/200              1 ....................-Q................. 39
```

Acidic & PxxP

```
HSIV-vif NL4-3    40 HGAITSSNTAANNAACAWLEAQEEEEVGFPVTPQVPLRPM 79
C/196             41 ........................................ 80
C/200             40 ........................................ 79
```

Core domain

```
NHSIV-vif NL4-3   80 TYKAAVDLSHFLKEKGGLEGLIHSQRRQDILDLWIYHTQG 119
C/196             81 ........................................ 120
C/200             80 ........................................ 119
```

C-loop

```
HSIV-vif NL4-3   120 YFPDWQNYTPGPGVRYPLTFGWCYKLVPVEPDKVEEANKG 159
C/196            121 ........................................ 160
C/200            120 .....T.................................. 159
```

C-terminus

```
HSIV-vif NL4-3   160 ENTSLLHPVSLHGMDDPEREVLEWRFDSRLAFHHVARELH 199
C/196            161 ...N.........I..........K............... 200
C/200            160 ...N..............K....................Y 199

HSIV-vif NL4-3   200  PEYFKNC 206
C/196            201  ....... 207
C/200            200  ....... 206
```

Envelope Sequences:

gp 120

```
HSIV-vif NL4-3    1 MRVKEKYQHLWRWGWKWGTMLLGILMICSATEKLWVTVYYGVPVWKEATTTLFCASDAKA 60
C/196             1 ............................................................ 60
C/200             1 ............................................................ 60

HSIV-vif NL4-3   61 YDTEVHNVWATHACVPTDPNPQEVVLVNVTENFNMWKNDMVEQMHEDIISLWDQSLKPCV 120
C/196            61 ............................................................ 120
C/200            61 ............................................................ 120
```

V1/V2

```
HSIV-vif NL4-3 121 KLTPLCVSLKCTDLKNDTNTNSSSGRMIMEKGEIKNCSFNISTSIRDKVQKEYAFFYKLD 180
C/196          121 ........................EG.T.................K.............. 180
C/200          121 ........................EG....E..............K.............. 180

HSIV-vif NL4-3 181 IVPIDNTSYRLISCNTSVITQACPKVSFEPIPIHYCAPAGFAILKCNNKTFNGTGPCTNV 240
C/196          181 ................................................E........... 240
C/200          181 .....................................................I...... 240

HSIV-vif NL4-3 241 STVQCTHGIRPVVSTQLLLNGSLAEEDVVIRSANFTDNAKTIIVQLNTSVEINCTRPNNN 300
C/196          241 ......................................V..................... 300
C/200          241 ......................................V..................... 300
```

V3

```
HSIV-vif NL4-3 301 TRKSIRIQRGPGRAFVTIGKIGNMRQAHCNISRAKWNATLKQIASKLREQFGNNKTIIFK 360
C/196          301 .......H.................................................... 360
C/200          301 .......H.................................................... 360
```

```
                                                       V4
HSIV-vif NL4-3 361 QSSGGDPEIVTHSFNCGGEFFYCNSTQLFNSTWFNSTWSTEGSNNTEGSDTITLPCRIKQ 420
C/196          361 ..........M................................................. 420
C/200          361 ............................................................ 420

                                                            V5
HSIV-vif NL4-3 421 FINMWQEVGKAMYAPPISGQIRCSSNITGLLLTRDGGNNNNGSEIFRPGGGDMRDNWRSE 480
C/196          421 ......K..................................................... 480
C/200          421 ......K..................................................... 480

                                       gp 120   gp 41
HSIV-vif NL4-3 481 LYKYKVVKIEPLGVAPTKAKRRVVQREKRAVGIGALFLGFLGAAGSTMGCTSMTLTVQAR 540
C/196          481 ........L........................................AA......... 540
C/200          481 ........L........................................AA......... 540

HSIV-vif NL4-3 541 QLLSDIVQQQNNLLRAIEAQQHLLQLTVWGIKQLQARILAVERYLKDQQLLGIWGCSGKL 600
C/196          541 ....G....................................................... 600
C/200          541 ....G....................................................... 600

HSIV-vif NL4-3 601 ICTTAVPWNASWSNKSLEQIWNNMTWMEWDREINNYTSLIHSLIEESQNQQEKNEQELLE 660
C/196          601 ............................................................ 660
C/200          601 ............................................................ 660

                                       Membrane spanning domain
HSIV-vif NL4-3 661 LDKWASLWNWFNITNWLWYIKLFIMIVGGLVGLRIVFAVLSIVNRVRQGYSPLSFQTHLP 720
C/196          661 .....................................................L...... 720
C/200          661 ............................................................ 720

HSIV-vif NL4-3 721 IPRGPDRPEGIEEEGGERDRDRSIRLVNGSLALIWDDLRSLCLFSYHRLRDLLLIVTRIV 780
C/196          721 ...........G.......................N........................ 780
C/200          721 ...........G................................................ 780

HSIV-vif NL4-3 781 ELLGRRGWEALKYWWNLLQYWSQELKNSAVNLLNATAIAVAEGTDRVIEVLQAAYRAIRH 840
C/196          781 ..............................................I............. 840
C/200          781 ............................................................ 840

HSIV-vif NL4-3 841 IPRRIRQGLERILL 854
C/196          841 .............. 854
C/200          841 ...........V.. 854
```

Vif sequences:

```
HSIV-vif NL4-3   1  MEEEKRWIAVPTWRIPERLERWHSLIKYLKYKTKDLQRVC 40
C/196            1  ........................................ 40
C/200            1  ........................................ 40

HSIV-vif NL4-3  41 YVPHHKVGWAWWTCSRVIFPLQEESQLEVQGYWNLTPERG 80
C/196           41 ........................................ 80
C/200           41 .....................E.-................ 79

HSIV-vif NL4-3  81 WLSTYAVRITWYSRNFWTDVTPDYADILLHSTYFPCFTAG 120
C/196           81 ........................................ 120
C/200           80 ........................................ 119

HSIV-vif NL4-3 121 EVRRAIRGEQLLSCCRFPRAHKNQVPSLQYLALRVVSYVR 160
C/196          121 ........................................ 160
C/200          120 .....T.................................. 159
```

```
HSIV-vif NL4-3 161 SQRENPTWKQWRRDNRRSLRMAKQNSRGDKQRGSKPPTKG 200
C/196          161 ...................I.................... 200
C/200          160 ...................I.................... 199

HSIV-vif NL4-3 201 ADFPGLAKVLGILA 214
C/196          201 .............. 214
C/200          200 .............. 213
```

Supplementary Fig. 6: Sequence alignment of biological isolates (C/196 and C/200). Nef, Vif, and Envelope protein sequences of biological isolates recovered PTM MO8009 are aligned to parental HSIV-vif_{NL4-3} sequences.

Envelope sequences:

```
HSIV-vifNL4-3  MRVKEKYQHLWRWGWKWGTMLLGILMICSATEKLWVTVYYGVPVWKEATTTLFCASDAKA
HSIV-P3-114    .....................P......................................
HSIV-P3-161    ............................................................
HSIV-P3-284    ............................................................

HSIV-vifNL4-3  YDTEVHNVWATHACVPTDPNPQEVVLVNVTENFNMWKNDMVEQMHEDIISLWDQSLKPCV
HSIV-P3-114    ............................................Q...........R...
HSIV-P3-161    ............................................Q...........R...
HSIV-P3-284    ............................................Q...........R...

HSIV-vifNL4-3  KLTPLCVSLKCTDLKNDTNTNSSSGRMIMEKGEIKNCSFNISTSIRDKVQKEYAFFYKLD
HSIV-P3-114    ........................EG.T.................K..............
HSIV-P3-161    ........................EG.T.................K..............
HSIV-P3-284    ........................EGTT.................K..............

HSIV-vifNL4-3  IVPIDNTSYRLISCNTSVITQACPKVSFEPIPIHYCAPAGFAILKCNNKTFNGTGPCTNV
HSIV-P3-114    ................................................E...........
HSIV-P3-161    ................................................E...........
HSIV-P3-284    ................................................E...........

HSIV-vifNL4-3  STVQCTHGIRPVVSTQLLLNGSLAEEDVVIRSANFTDNAKTIIVQLNTSVEINCTRPNNN
HSIV-P3-114    ......................................V.....................
HSIV-P3-161    ......................................V.....................
HSIV-P3-284    ......................................V.....................

HSIV-vifNL4-3  TRKSIRIQRGPGRAFVTIGKIGNMRQAHCNISRAKWNATLKQIASKLREQFGNNKTIIFK
HSIV-P3-114    .......H................................R...................
HSIV-P3-161    .......H................................R...................
HSIV-P3-284    .......H................................R....E..............

HSIV-vifNL4-3  QSSGGDPEIVTHSFNCGGEFFYCNSTQLFNSTWFNSTWSTEGSNNTEGSDTITLPCRIKQ
HSIV-P3-114    ..........M.................................................
HSIV-P3-161    ..........M.................................................
HSIV-P3-284    ..........M.................................................

HSIV-vifNL4-3  FINMWQEVGKAMYAPPISGQIRCSSNITGLLLTRDGGNNNNGSEIFRPGGGDMRDNWRSE
HSIV-P3-114    ......K.....................................................
HSIV-P3-161    ......K.....................................................
HSIV-P3-284    ......K.....................................................

HSIV-vifNL4-3  LYKYKVVKIEPLGVAPTKAKRRVVQREKRAVGIGALFLGFLGAAGSTMGAASMTLTVQAR
HSIV-P3-114    ........L...................................................
HSIV-P3-161    ........L...................................................
HSIV-P3-284    ........L...................................................

HSIV-vifNL4-3  QLLSDIVQQQNNLLRAIEAQQHLLQLTVWGIKQLQARILAVERYLKDQQLLGIWGCSGKL
HSIV-P3-114    ....G..............................T........................
HSIV-P3-161    ....G..............................T........................
HSIV-P3-284    ....G..............................T........................

HSIV-vifNL4-3  ICTTAVPWNASWSNKSLEQIWNNMTWMEWDREINNYTSLIHSLIEESQNQQEKNEQELLE
HSIV-P3-114    ............................................................
HSIV-P3-161    ............................................................
HSIV-P3-284    ............................................................

HSIV-vifNL4-3  LDKWASLWNWFNITNWLWYIKLFIMIVGGLVGLRIVFAVLSIVNRVRQGYSPLSFQTHLP
HSIV-P3-114    ............................................................
HSIV-P3-161    ............................................................
HSIV-P3-284    ............................................................

HSIV-vifNL4-3  IPRGPDRPEGIEEEGGERDRDRSIRLVNGSLALIWDDLRSLCLFSYHRLRDLLLIVTRIV
```

HSIV-P3-114 ...........G.......................N.......................G
HSIV-P3-161 ...........G.......................N.......................G
HSIV-P3-284 ...........G.......................N.......................G

HSIV-vif$_{NL4-3}$ ELLGRRGWEALKYWWNLLQYWSQELKNSAVNLLNATAIAVAEGTDRVIEVLQAAYRAIRH
HSIV-P3-114 ..........................S...................I.............
HSIV-P3-161 ....I.............
HSIV-P3-284 ....I.............

HSIV-vif$_{NL4-3}$ IPRRIRQGLERILL
HSIV-P3-114 ....
HSIV-P3-161 ....
HSIV-P3-284 ....

Nef sequences:

HSIV-vif$_{NL4-3}$ MGGKWSKSSVIGWPAVRERMRRAEPAADGVGAVSRDLEKHGAITSSNTAANNAACAWLEA
HSIV-P3-114 ....
HSIV-P3-161 ....T.......
HSIV-P3-284 ....

HSIV-vif$_{NL4-3}$ QEEEEVGFPVTPQVPLRPMTYKAAVDLSHFLKEKGGLEGLIHSQRRQDILDLWIYHTQGY
HSIV-P3-114 ....K...............
HSIV-P3-161 ....K...............
HSIV-P3-284 ....K...............

HSIV-vif$_{NL4-3}$ FPDWQNYTPGPGVRYPLTFGWCYKLVPVEPDKVEEANKGENTSLLHPVSLHGMDDPEREV
HSIV-P3-114 ....N.........I......M
HSIV-P3-161 ....N.........I.......
HSIV-P3-284 ....N.........I.......

HSIV-vif$_{NL4-3}$ LEWRFDSRLAFHHVARELHPEYFKNC
HSIV-P3-114 ...K................D.....
HSIV-P3-161 ...K................D.....
HSIV-P3-284 ...K................D.S...

Rev Sequences:

HSIV-vif$_{NL4-3}$ MAGRSGDSDEELIRTVRLIKLLYQSNPPPNPEGTRQARRNRRRRWRERQRQIHSISERIL
HSIV-P3-114 ....
HSIV-P3-161 ....
HSIV-P3-284 ....

HSIV-vif$_{NL4-3}$ STYLGRSAEPVPLQLPPLERLTLDCNEDCGTSGTQGVGSPQILVESPTVLESGTKE*
HSIV-P3-114 ....E.......................R......R.....................
HSIV-P3-161 ....E.......................R......R.....................
HSIV-P3-284 ....E.......................R......R.....................

Supplementary Fig. 7: Sequence alignment of HSIV-P3 IMCs. Envelope, Nef, and Rev protein sequences of HSIV-P3 IMCs are aligned to parental HSIV-vif$_{NL4-3}$ sequences.

```
                                                    Splice acceptor site
HSIV-vifNL4-3            TTTTACTGCATAGCACTTATTTCCCTTGCTTTACAGCGGGAGAAGTGAGA
Vpr+HSIV clones          ..................................................
HSIV-P3-114              .......A..........................................
HSIV-P3-161              .......A..........................................
HSIV-P3-284              .......A..........................................

HSIV-vifNL4-3            AGGGCCATCAGGGGAGAACAACTGCTGTCTTGCTGCAGGTTCCCGAGAGC
Vpr+HSIV clones          ..................................................
HSIV-P3-114              ....................G.............................
HSIV-P3-161              ....................G.............................
HSIV-P3-284              ....................G.............................

HSIV-vifNL4-3            TCATAAGAACCAGGTACCAAGTCTACAGTACTTAGCACTGAGAGTAGTAA
Vpr+HSIV clones          ..................................................
HSIV-P3-114              ..................................................
HSIV-P3-161              ..................................................
HSIV-P3-284              ..................................................
                            Vpx start codon
HSIV-vifNL4-3            GTTATGTCAGATCCCAGAGAGAGAATCCCACCTGGAAACAGTGGAGAAGA
Vpr+HSIV clones          ...ACG............................................
HSIV-3-114               ...ATG............................................
HSIV-P3-161              ...ATG............................................
HSIV-P3-284              ...ATG............................................
                                             Met codon
HSIV-vifNL4-3            GACAATAGGAGAAGCCTTCGAATGGCTAAACAGAACAGTAGAGGAGATAA
Vpr+HSIV clones          .....................TTG..........................
HSIV-P3-114              .....................ATA..........................
HSIV-P3-161              .....................ATA..........................
HSIV-P3-284              .....................ATA..........................

HSIV-vifNL4-3            ACAGAGAGGCAGTAAACCACCTACCAAGGGAGCTGATTTTCCAGGTTTGG
Vpr+HSIV clones          ..................................................
HSIV-P3-114              ..................................................
HSIV-P3-161              ..................................................
HSIV-P3-284              ..................................................
                                                            Deletion
HSIV-vifNL4-3            CAAAGGTCTTGGGAATACTGGCATGATGAGTTAGGAAACTGACAGAGGAC
Vpr+HSIV clones          ......................T.--------------------------
HSIV-P3-114              ......................T.--------------------------
HSIV-P3-161              ......................T.--------------------------
HSIV-P3-284              ......................T.--------------------------
                           Vpr start codon
HSIV-vifNL4-3            AGATGGAACAAGCCCCAGAAGACCAAGGGCC
Vpr+HSIV clones          ...............................
HSIV-P3-114              ...............................
HSIV-P3-161              ...............................
HSIV-P3-284              ...............................
```

Supplementary Fig. 8: Sequence alignment of SIV vif and HIV-1 vpr region of HSIV-P3 IMCs with Vpr^+ and Vpr- HSIV-vif_{NL4-3}.

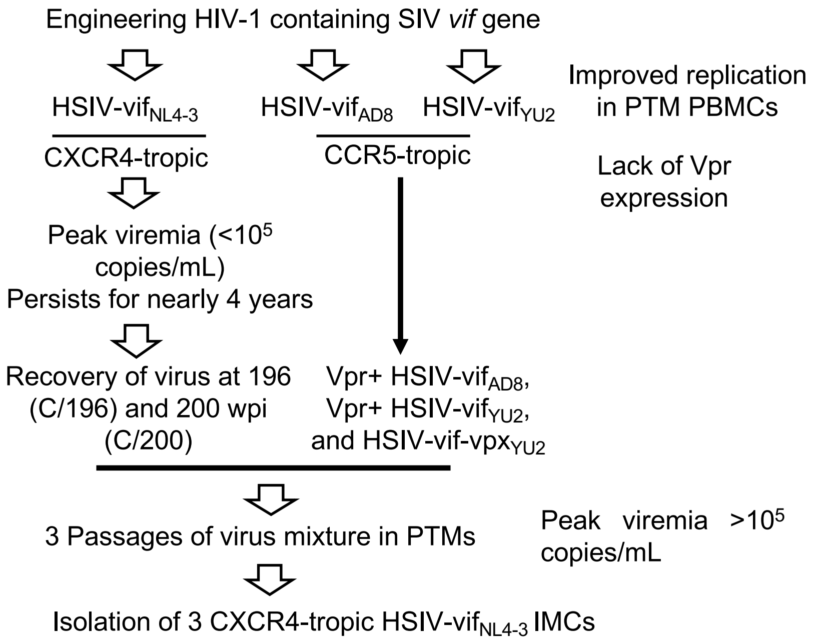

Supplementary Fig 9: Flowchart showing overall experimental plan to recover HSIV infectious molecular clones.
